# Ascertaining the Career Intentions of Medical Students (AIMS) in the United Kingdom Post Graduation: Protocol for a Mixed Methods Study

**DOI:** 10.2196/45992

**Published:** 2023-06-19

**Authors:** Tomas Ferreira, Alexander M Collins, Rita Horvath

**Affiliations:** 1 School of Clinical Medicine University of Cambridge Cambridge United Kingdom; 2 School of Public Health, Faculty of Medicine Imperial College London London United Kingdom

**Keywords:** medical students, NHS, National Health Service, career intent, attitude, opinion, workforce, workforce planning, medical education, career, doctor, medical training, medical graduate, cross-sectional, thematic analysis, degree, rotation

## Abstract

**Background:**

Among doctors in the United Kingdom, there is growing sentiment regarding delaying specialist training, emigrating to practice medicine abroad, or leaving the profession altogether. This trend may have substantial implications for the future of the profession in the United Kingdom. The extent to which this sentiment is also present in the medical student population is not well understood.

**Objective:**

Our primary outcome is to determine current medical students’ career intentions after graduation and upon completing the foundation program and to establish the motivations behind these intentions. Secondary outcomes include determining which, if any, demographic factors alter the propensity to pursue different career paths available to a medical graduate, determining which specialties medical students plan on pursuing, and understanding current views on the prospect of working in the National Health Service (NHS).

**Methods:**

The Ascertaining the Career Intentions of Medical Students (AIMS) study is a national, multi-institution, and cross-sectional study in which all medical students at all medical schools in the United Kingdom are eligible to participate. It was administered via a novel, mixed methods, and web-based questionnaire and disseminated through a collaborative network of approximately 200 students recruited for this purpose. Both quantitative and thematic analyses will be performed.

**Results:**

The study was launched nationally on January 16, 2023. Data collection was closed on March 27, 2023, and data analysis has commenced. The results are expected to be available later in the year.

**Conclusions:**

Doctors’ career satisfaction within the NHS is a well-researched topic; however, there is a shortage of high-powered studies that are able to offer insight into medical students’ outlook on their future careers. It is anticipated that the results of this study will bring clarity to this issue. Identified areas of improvement in medical training or within the NHS could be targeted to improve doctors’ working conditions and help retain medical graduates. Results may also aid future workforce-planning efforts.

**International Registered Report Identifier (IRRID):**

DERR1-10.2196/45992

## Introduction

### Background

In the United Kingdom, medical degrees typically last for 5 or 6 years, depending on whether one elects to pursue an additional year in the awarding of an intercalated degree. Alternatively, for previous degree holders, there is a 4-year accelerated medicine course. Upon the completion of their medical degree, graduates are provisionally registered with the General Medical Council (GMC). Graduates then enter the mandatory 2-year United Kingdom Foundation Programme (UKFP), consisting of rotations in various settings and specialties. After the first year of the UKFP, graduates are awarded full medical registration with the GMC. Doctors who have completed the UKFP are then able to pursue further training, which may be in very specialized fields, for example, ophthalmology or neurosurgery, or broader pathways such as general practice or internal medicine training [1].

Historically, the vast majority of UKFP graduates pursued specialty training immediately after completing their foundation program; for instance, in 2010, the proportion of graduates entering further training upon completing Foundation Year 2 was 83.1%. However, the UKFP Office career destination report revealed that this number had fallen to only 34.9% of doctors in 2019 [2]. The UKFP Office has not repeated the survey since, so it is difficult to surmise how these statistics may have changed in the interim.

In comparison to most other Organization for Economic Co-operation and Development countries, the United Kingdom has fewer doctors per capita [3]. One of the ways the British government has attempted to combat this issue is through the opening of new medical schools as well as increasing student capacity in existing medical schools [4,5]. The aim of the Ascertaining the Career Intentions of Medical Students (AIMS) study is to understand the effectiveness of this approach: are the factors encouraging graduate doctors to seek alternatives to medicine in the United Kingdom, or in its entirety, also present in the undergraduate population?

AIMS also endeavors to investigate the reasons behind students’ intentions to both enter and leave the standard training pathway and attempt to identify any demographic and background information that might increase the propensity to leave training.

A British Medical Association survey of 8000 senior doctors determined that 44% of National Health Service (NHS) consultants in England plan to leave or take a break from working in the NHS over the next year [6]. Similarly, a recent survey of 4553 junior doctors in the NHS reported that 4 in 10 plan to leave the NHS, with 33% of these wanting to immigrate to another country to work [7]. Broader literature supports these findings, with a 40-year survey series of junior doctors highlighting that the desire to remain in UK medical practice was lower in 2015 than had ever been recorded [8]. Additionally, in 2021, nearly 10,000 doctors relinquished their license to practice in the United Kingdom, making up nearly 1 out of 10 of the total UK doctor workforce [5,9]. We aim to determine if this trend, found in both junior and senior doctors alike, may also manifest in the intentions of medical students yet to even enter the profession.

The study invites participants to share their views on many aspects of working in the NHS and how the prospect of working in the NHS could be improved. Data collected from this study could prove helpful in addressing some of the concerns of those making up the future of the profession. In Australia and New Zealand, a questionnaire is distributed each year where the medical deans of medical schools in the country survey final-year medical students and collect data on their background, medical school experience, and any early geographical and specialty preferences for their future career [10]. The information gained has had a lasting positive impact on curriculum development and future medical workforce planning. Additionally, it has assisted in workforce distribution and helped to plan specialist training pathways to meet the career interests of trainees and the public’s health needs.

The AIMS study is a national, multi-institutional, and cross-sectional study. AIMS endeavors to understand the factors involved in medical students’ decision-making around their future careers. Specifically, we are interested in students’ current career plans and why they may or may not intend to pursue specialty training or a medical career in the United Kingdom. We are also hoping to understand current views on the prospect of working in the NHS.

### Study Objectives

#### Primary Objectives

This primary objectives are to determine current medical students’ career intentions after graduation and upon completing the foundation program and to ascertain the motivations behind these intentions.

#### Secondary Objectives

Secondary objectives are as follows:

- To characterize the backgrounds of medical students intending to:
  emigrate to practice abroad
  leave medicine permanently
  undertake further study or a career break
  not going into further medical training immediately after degree completion or foundation training
- To determine which specialties medical students plan on pursuing
- To analyze medical students’ views on how the prospect of working in the NHS could be improved

## Methods

### Study Design

AIMS is a national, multi-institutional, and cross-sectional study of medical students. Participants’ responses will be recorded via a web-based survey platform. The survey contains 71 items, but participants will not view all of them as their visibility depends on their previous answers. The shortest question sequence involves 30 items, and the longest involves 43 items. Questions are structured using a combination of Likert scale matrices, multiple-choice options, and free-text entry to broaden the capture of sentiment nuance and improve precision in the data. Data collection began on January 16, 2023, and was completed on March 27, 2023. A full project timeline can be found in Table 1. This timeline will be approached with a level of flexibility, such as for centers with logistical obstacles to data collection.

**Table 1:** Project timeline.

Dates	Activity
December 12, 2022, to January 9, 2023	Recruitment of collaborators throughout all UK medical schools via social media and communication with medical schools and societies
January 10, 2023, to January 15, 2023	Training of collaborators
January 16, 2023, to March 27, 2023	Study runs nationally for approximately 2 months
March 28, 2023, to June 1, 2023	Data analysis and manuscript preparation

The survey consists of multiple sections. Section 1 of the survey involves a background and demographic section, which all participants will answer. Consent is also obtained in this section. Section 2 focuses on participants’ career intentions immediately after graduation and after foundation training (for those entering it). Section 3 invites participants to explore the factors behind their decision-making. Section 4 surveys which specialties the participants are currently inclined to pursue. The final section includes a free-entry text box where participants are invited to articulate how the prospect of working in the NHS could be improved and a question to obtain consent for follow-up studies.

The complete 71-item questionnaire can be found in Multimedia Appendix 1.

To identify gaps in knowledge and inform the aim of the project, a review of existing literature was conducted, including similar questionnaires and qualitative studies on students’ and doctors’ perspectives. Feedback from senior clinical staff was also obtained. Questions were revised based on the comments received, aiming to ensure they were as nondirective and comprehensive as possible while remaining unencumbering so as to maximize the number of responses for statistical power.

The survey will be hosted on the Qualtrics survey platform (Qualtrics XM), a General Data Protection Regulation (GDPR)–compliant web-based survey platform that supports both mobile and desktop devices. The survey will be distributed through university mailing lists, student society social media pages, conferences, and personal and medical school social media platforms (such as Facebook, Instagram, LinkedIn, and Twitter). To maximize distribution across the United Kingdom, a national network of approximately 200 AIMS Collaborative members was recruited, representing all medical schools in the United Kingdom. Each member will be asked to liaise with their medical school dean to distribute the survey through official channels and advertise it at regular intervals over the collection period from January to March.

### Study Population

All current students in all years at UK medical schools recognized by the GMC and the Medical Schools Council are eligible to participate in the study. A list of eligible medical schools and approved programs can be found in Multimedia Appendix 2. It is worth noting that we have excluded schools that, although approved by the GMC, have not yet admitted their first cohort of students since they have no medical students to participate.

### Data Collection

The survey will be disseminated primarily through medical student collaborators recruited prior to the study launch. These collaborators will ensure that their medical school is formally engaged at an early stage of this study and will be primarily responsible for disseminating this questionnaire among students at their medical school.

### Ethics Approval

Ethical approval was granted by the University of Cambridge Research Ethics Committee (reference PRE.2022.124) on January 5, 2023.

To obtain informed consent, a participant information sheet (Multimedia Appendix 3) will be made available to all participants. This information sheet will explain the rationale and purpose of the study, emphasizing its voluntary nature and the anonymity and confidentiality of participants. It will be made clear on the study materials and on the first page of the questionnaire that submitting responses provides consent for data usage. Additionally, the first question of the survey will obtain consent, and it will be mandatory to complete. Consent can be withdrawn at any time, during or after completing the questionnaire, by contacting the study lead. Email addresses will only be collected and stored for participants who provide explicit consent to this in the final question of the survey. Email addresses will be kept separate from the responses to survey questions, ensuring anonymity.

The anonymous responses will be collected and stored on the secure web-based server, Qualtrics. Survey data will be extracted from Qualtrics and stored in a password-protected Microsoft Excel (version 16.71; Microsoft) file accessible only to the central study team. The University of Cambridge standards for data handling will be followed for all data management and record-keeping.

Participants will not receive compensation for completing the questionnaire. However, participants who provide their contact information will be entered into a prize draw, with one randomly selected individual receiving a cash prize of £300 (US $370.10) at the time of prize draw, at the end of data collection.

### Data Analysis

A sample size calculation was performed, and it was determined that a minimum of 8026 participants are needed to have a confidence level of 95% that the results of the survey are within 1% representation of the total medical student population. This calculation used a population size for UK medical students acquired via a Freedom of Information request to the GMC.

The study will adhere to the STROBE (Strengthening the Reporting of Observational Studies in Epidemiology) guidelines for cross-sectional studies [11]. Descriptive statistics and comparisons between student year groups and other participant characteristics will be used. Appropriate statistical tests will be applied for these comparisons, such as the chi-square test (for categorical variables) and Student *t* test (for continuous variables). For free-text questions yielding qualitative data, a thematic analysis will be conducted using the reflexive approach of Braun and Clarke [12]. Data analysis will be performed using Microsoft Excel and RStudio (version 4.2.1; RStudio, PBC).

## Results

Following study completion, teleconferences will be conducted with all collaborators to share and discuss the data analysis undertaken and the study results. Following this, it is intended that results will be presented at local, regional, national, and international conferences. In addition, the results will be disseminated via publication in a peer-reviewed medical journal. All collaborators will be given PubMed-citable collaborative coauthorship under the institutional name “the AIMS Collaborative”. We will have a hybrid authorship list of named authors and the institutional collaborative.

Following study completion, medical school collaborators will be able to request their institution-specific data and the analysis performed on said data from the AIMS steering committee. This data will be anonymized. The fully anonymized data set will be made publicly available.

Data collection is currently underway, with the study having launched nationally on January 16, 2023. Data collection was closed on March 27, 2023, and data analysis has commenced. The results are expected to be available later in the year.

## Discussion

### Previous Literature

Although there have been (1) studies exploring which specialties junior doctors or medical students intend on pursuing [13-18], (2) studies that focused on career intentions of those pursuing one specialty or exploring factors that attract them to one specific specialty [19-35], (3) studies specifically focused on reasons why doctors are leaving the United Kingdom [36-40], (4) studies exploring how medical students and junior doctors feel about specific aspects of working within the NHS [41-44], and (5) studies investigating the desire for a career break post Foundation Year 2 (also known as the “F3”) [45-47], there have been no recent, high-powered studies explicitly aimed at medical students, irrespective of current career ambitions or seniority, investigating overall career intentions and correlating it with demographic factors and medical student seniority. There has been one similar study, although it focuses entirely on medical students and their intentions to leave the NHS, which is limited by its low power and lack of subanalysis regarding student seniority and demographic factors [48]. We aim to address these limitations. Additionally, this study will be the first of a series of follow-up surveys of medical students, thus being the first longitudinal study of medical students’ career intentions of its kind.

### Strengths

Owing to its rigorous collaborator recruitment plans with the aim of high participant recruitment, this study will provide an accurate representation of medical students’ intentions regarding practicing in the United Kingdom or abroad, as well as their preferred specialties. This information can be valuable for workforce planning.

The study will determine the current undergraduate consensus on several aspects of working in the NHS, which could help future policymakers prioritize change and better prepare medical schools’ junior students for the reality of such aspects.

This study will determine which, if any, of the included demographic factors alter the propensity to pursue different career paths available to a medical graduate, providing insights into potential disparities.

### Limitations

The study is limited by its snapshot nature, as it does not capture changes in career intentions over time. However, it is our intention to follow up with consenting participants and determine how their career intentions may have changed.

It is possible that those already intending to exit the profession, emigrate, or are dissatisfied with the current circumstances will be more likely to complete the survey. To mitigate this, we have recruited approximately 200 collaborators who will distribute the survey in their respective medical schools, targeting all medical students. Additionally, the promotional material and the questions have been written not to imply any particular outcome is predicted or sought.

Challenges in recruiting collaborators or ensuring sufficient student responses may impact the achievement of the study’s national goals.

The study’s response options for each question may be limited, potentially affecting the comprehensiveness of the data. However, an extensive literature review, peer survey testing, and input from senior clinicians provide confidence that the survey will capture the majority of students’ career intentions and opinions. Additionally, a free-text box allows participants to express their views more freely.

This study presents a unique opportunity to accurately assess how medical students view their current career prospects and assess the feasibility of potentially implementing a regular, census-like survey of UK medical students to assist the government’s efforts in workforce planning and addressing grievances within the workforce, therefore resulting in higher job satisfaction.

## Appendix

Multimedia Appendix 1 AIMS (Ascertaining the Career Intentions of Medical Students) survey.

Multimedia Appendix 2 List of eligible medical schools.

Multimedia Appendix 3 Participant Information Sheet.

## Abbreviations

AIMS: Ascertaining the Career Intentions of Medical Students
GDPR: General Data Protection Regulation
GMC: General Medical Council
NHS: National Health Service
STROBE: Strengthening the Reporting of Observational Studies in Epidemiology
UKFP: United Kingdom Foundation Programme

## Data Availability

The data sets generated and analyzed during this study will be made publicly available once the study is completed.
